# Robotic Partial Nephrectomy with En Bloc Removal of a Renal Vein Thrombus for Multiple cT3a Renal Cell Carcinoma Lesions

**DOI:** 10.1016/j.euros.2022.08.002

**Published:** 2022-08-22

**Authors:** Antonio Andrea Grosso, Diego Marcos Marìn, Fabrizio Di Maida, Maria Lucia Gallo, Luca Lambertini, Samuele Nardoni, Andrea Mari, Andrea Minervini

**Affiliations:** a Department of Experimental and Clinical Medicine, University of Florence-Unit of Oncologic Minimally Invasive Urology and Andrology, Careggi Hospital, Florence, Italy; b Hospital Universitario Puerta de Hierro Majadahonda, Madrid, Spain

**Keywords:** Cognitive surgery, Partial nephrectomy, Renal cell carcinoma, Robotic, Three-dimensional models

## Abstract

Partial nephrectomy (PN) may be recommended for selected patients with advanced-stage (>cT2) renal cell carcinoma (RCC) with the aim of avoiding dialysis and chronic kidney disease–related comorbidities. The spread of robotic surgery has led to expansion of PN indications to more challenging scenarios and even frontier surgeries, including advanced-stage RCC. Here we describe the management of a patient with a solitary kidney diagnosed with multiple cT3a (renal vein thrombus) RCC who was treated using a conservative robotic approach. The most crucial surgical considerations for this procedure were: (1) tailored planning of the surgical approach using three-dimensional reconstruction software; (2) accurate boundary delineation for the tumors and thrombus; (3) avoiding unnecessary warm ischemia time; (4) performing an anatomical excision to follow eventual tumor bulging; and (5) en bloc removal of the main lesion and its thrombus. No perioperative complications were recorded. Histopathology revealed clear cell RCC for all four lesions with nucleolar grade 3 and negative surgical margins. At 12-mo follow-up the patient was disease-free. When performed by an experienced surgeon, PN plus venous thrombus excision for imperative cases with cT3 RCC may represent a valid treatment option with valuable oncological and functional outcomes.

**Patient summary:**

We describe the case of patient who had a single kidney with multiple kidney tumors and tumor extension into a blood vessel. The patient was treated with robot-assisted removal of the tumors, sparing as much kidney tissue as possible. This technique was found to be safe and effective, with no complications and good intermediate-term results.

## Take Home Message

When performed by an experienced surgeon, robotic partial nephrectomy plus venous thrombus excision may represent as valid treatment option for imperative indications, with valuable oncological and functional outcomes. Specific surgical nuances are necessary to effectively conduct such a procedure.

## 1 Background

Despite the increasing diagnosis of renal masses in earlier stages of disease, up to 5% of renal cell carcinomas (RCCs) present at an advanced stage, including renal vein invasion with tumor thrombus with or without caval extension [1]. Owing to the higher risk of recurrence and cancer-specific death in this subgroup, radical nephrectomy (RN) with thrombus excision is highly warranted for elective indications [1]. However, for some imperative conditions including solitary kidneys and preoperative impaired renal function, partial nephrectomy (PN) for locally advanced stages may be considered with the aim of avoiding dialysis and chronic kidney disease (CKD)-related comorbidities [2].

In cT3a RCC with no evidence of systemic disease, imperative PN yielded promising results [3]. A recent matched-pair analysis comparing imperative PN versus RN for RCC with tumor thrombus showed no significative differences in terms of 5-yr overall survival and cancer-specific survival even though the PN cohort experienced a higher rate of surgery-related complications and transfusions [3]. These findings led to debate regarding the adoption of PN in such a scenario between sceptics who prioritize the oncological safety and lower morbidity of a radical surgery and supporters favoring the functional-related benefits in cases with conservative intent.

In recent years, technological innovations and increasing experience among kidney cancer surgeons have progressively led to expansion of the adoption of PN to more challenging scenarios and even frontier surgeries. In particular, use of robotic assistance allows very precise tumor excision and was found to be protective in terms of acute kidney injury onset, postoperative complications, and failure to achieve trifecta outcomes when compared to open and laparoscopic PN [4]. In this scenario, the introduction of new tools to enhance preoperative evaluation of a tumor and its anatomical complexity plays a pivotal role. In this regard, CT images may be not entirely appropriate in tailoring the surgical strategy for complex renal masses since they rely on a static evaluation of tumor characteristics. Use of three-dimensional (3D) reconstructions for complex cases can improve assessment of the tumor anatomy, permitting better-informed surgical planning and potentially increasing the chances of achieving a “successful” surgery [5].

Based on these considerations, the present report describes the effective management strategy for a patient with a solitary kidney diagnosed with multiple cT3a RCC lesions and treated with a conservative approach.

## 2 Case presentation

A 61-yr-old man was referred to our institution after incidental diagnosis of multiple renal tumors located in his left solitary kidney. The patient had already undergone open right RN for a pT1a RCC nearly two decades earlier.

**Fig. 1:**
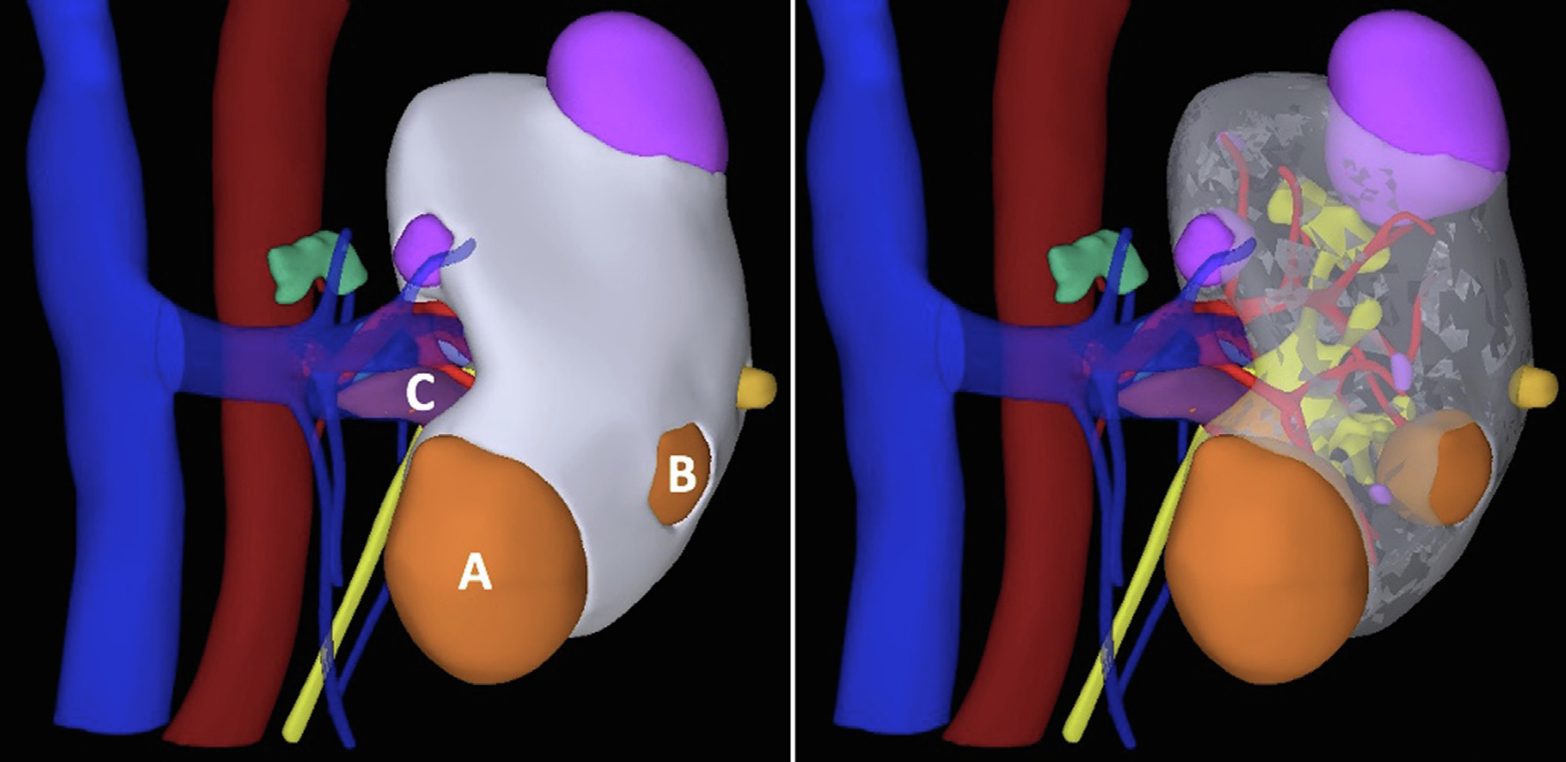
Three-dimensional virtual model showing (A) the main tumor located anteriorly in the lower kidney pole, (B) a secondary lesion, and (C) the venous tumor thrombus.

A computed tomography (CT) scan showed two renal masses. The main tumor was a 5-cm lesion in the anterior lower kidney pole with a tumor thrombus at the level of the inferior venous branch. The second tumor measured 3 cm in its largest diameter and was in close proximity to the main renal mass (Fig. 1). The patient’s serum creatinine level was 1.17 mg/dl (CKD stage II according to the Kidney Disease Improving Global Outcomes [KDIGO] classification).

**Fig. 2:**
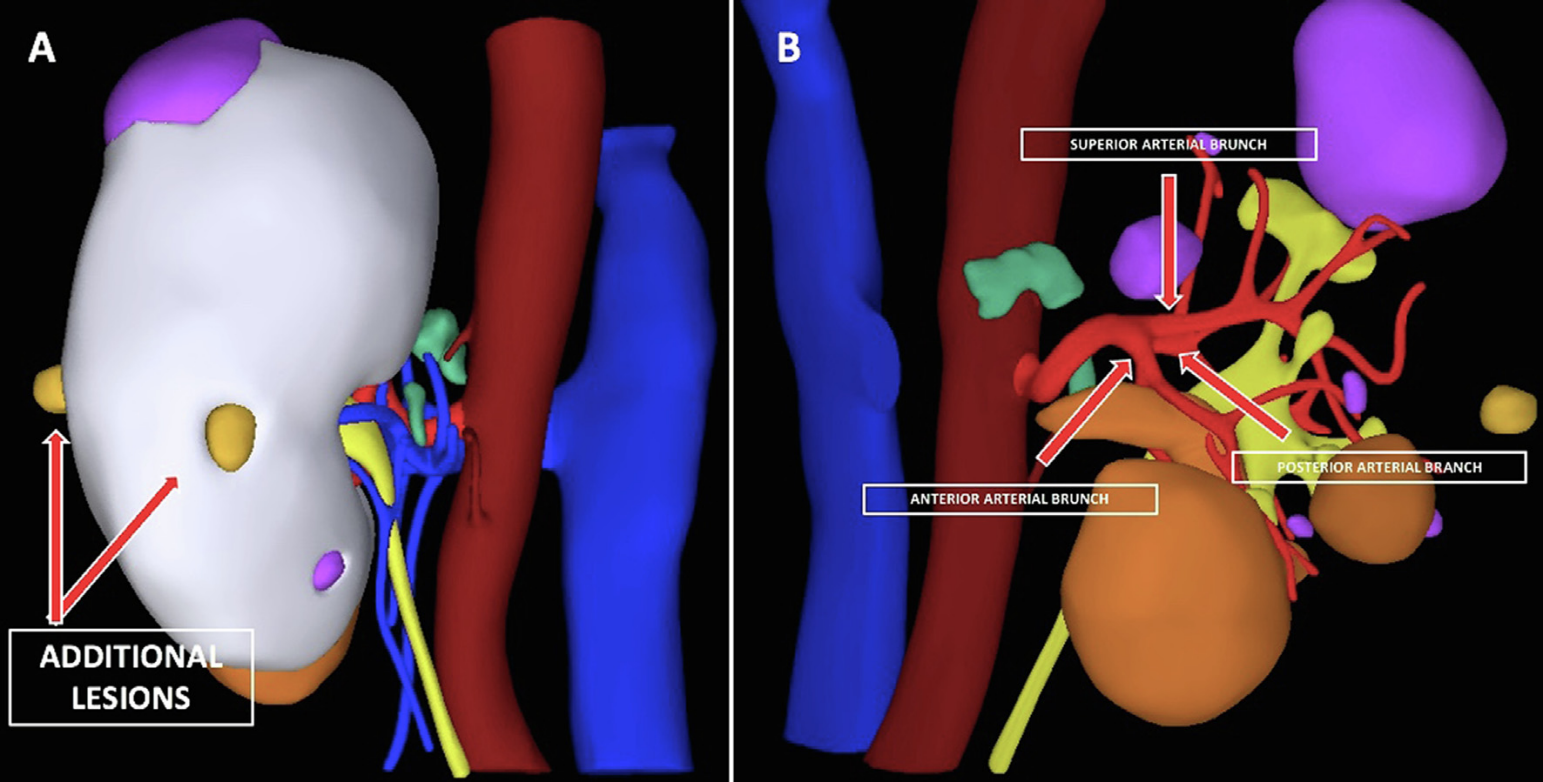
Three-dimensional virtual model showing (A) two small additional mediorenal lesions and (B) the main renal artery dividing into three collateral branches.

A tailored preoperative assessment was performed using dedicated software (M3DICS, Turin, Italy) to create a 3D virtual reconstruction from DICOM images from the CT scan, which significantly enhanced perception of the tumor anatomy and kidney vascularization. Interestingly, the 3D model revealed two additional small mediorenal lesions (Fig. 2A) and showed the main renal artery dividing into three collateral branches (Fig. 2B).

After multidisciplinary discussion, robot-assisted PN with tumor thrombus excision was indicated.

## 3 Surgical approach

A transperitoneal three-arm configuration was used. After identifying the renal pedicle, the main renal vein and artery with its collateral branches were individually dissected and isolated. The whole kidney was exposed by removing its fat covering to correctly identify the lesions and allow proper renal mobilization. The presence of two additional lesions identified on scouting with the 3D model was confirmed via intraoperative ultrasonography.

Surgical management began by addressing the three minor lesions via a clampless strategy. This choice was driven by the need to save minutes of ischemia time for the main part of the procedure. We conducted enucleoresection for the two small cortical lesions outlined by the 3D reconstruction and pure enucleation for the third lesion, since it exhibited prevalent endophytic growth.

In fact, while the excision strategy does not substantially affect the outcomes for cortical masses, widespread evidence has revealed that a pure enucleation approach increases the possibility of achieving trifecta outcomes [6] since following the natural cleavage plane between the kidney and the tumor surface maximizes nephron preservation while reducing the risk of positive surgical margins [7]. This strategy should be even more advisable for tumors with intrarenal growth in order to enhance exposure of potential tumor bulging.

Therefore, the main tumor contours were delineated using intraoperative ultrasound. A robotic linear probe can be handled independently by the surgeon, increasing the autonomy in achieving difficult angles while maintaining perpendicular contact with the kidney surface. This tool ameliorates the perception of the tumor burden, favors correct identification of the surgical plane, and allows assessment of possible interaction between the tumor and the caliceal system.

After incising the renal parenchyma a few millimeters away from the lesion, the collateral arterial branches were clamped. The correct cleavage plane was then identified and followed predominantly via blunt dissection. Pointwise hemostasis was performed to maintain a bloodless surgical field. In this step, total ischemia management involved clamping the main renal artery. When feasible, this “hybrid” ischemia strategy—starting the excision with selective clamping and deferring global ischemia for the inner enucleation field—reduces devascularization damage to the healthy parenchyma while limiting bleeding during the first steps of the excision.

The tumor was approached circumferentially in order to reduce possible tension. At this point, two Hem-o-Lok clips were placed at the level of the inferior venous branch and the tumor thrombus was excised en bloc with the mass. Renorraphy was performed by suturing the medulla and cortex separately using 2-0 Monosyn and 2-0 Vicryl sutures, respectively. The surgical procedure is demonstrated in the video in the Supplementary material.

Overall, the operative time was 168 min and the global ischemia time was 9 min. No perioperative complications were recorded. The estimated blood loss was 450 ml and the decrease in hemoglobin was 2.7 mg/dl on postoperative day 3. The patient was discharged on postoperative day 5. Histopathology revealed clear cell RCC for all four lesions, with nucleolar grade 3 and negative surgical margins. At 12-mo follow-up the patient was disease-free with a serum creatinine level of 1.8 mg/dl.

## 4 Conclusions

When performed by an experienced surgeon, robotic PN plus venous thrombus excision may represent a valid treatment option for imperative indications, with valuable oncological and functional outcomes. Further prospective studies are needed to validate our preliminary experience.

  ***Author contributions***: Andrea Minervini had full access to all the data in the study and takes responsibility for the integrity of the data and the accuracy of the data analysis.

*Study concept and design*: Minervini, Grosso.

*Acquisition of data*: Grosso, Marìn, Di Maida, Lambertini.

*Analysis and interpretation of data*: Grosso, Marìn.

*Drafting of the manuscript*: Grosso.

*Critical revision of the manuscript for important intellectual content*: Minervini, Mari.

*Statistical analysis*: None.

*Obtaining funding*: None.

*Administrative, technical, or material support*: Mari, Di Maida, Gallo, Nardoni.

*Supervision*: Minervini.

*Other*: None.

  ***Financial disclosures:*** Andrea Minervini certifies that all conflicts of interest, including specific financial interests and relationships and affiliations relevant to the subject matter or materials discussed in the manuscript (eg, employment/affiliation, grants or funding, consultancies, honoraria, stock ownership or options, expert testimony, royalties, or patents filed, received, or pending), are the following: None.

  ***Funding/Support and role of the sponsor:*** None.
